# (3*Z*,3′*Z*)-3,3′-(Ethane-1,2-diyl­idene)­bis[isobenzofuran-1(3*H*)-one]

**DOI:** 10.1107/S1600536809030888

**Published:** 2009-08-08

**Authors:** Katsuhiko Ono, Osamu Tokura, Masaaki Tomura

**Affiliations:** a Department of Materials Science and Engineering, Nagoya Institute of Technology, Gokiso, Showa-ku, Nagoya 466-8555, Japan; b Institute for Molecular Science, Myodaiji, Okazaki 444-8585, Japan

## Abstract

The title compound, C_18_H_10_O_4_, has been isolated as an impurity in commercially available 6,11-dihydr­oxy-5,12-naphth­acenedione. The title compound exhibits yellow fluorescence in the solid state. The mol­ecule has crystallographic inversion symmetry and is planar, with an r.m.s. deviation of 0.031 (1) Å. The crystal structure is stabilized by C—H⋯O hydrogen bonds and π–π stacking inter­actions between 3-methyl­eneisobenzofuran-1(3*H*)-one units [inter­planar distance 3.43 (1) Å].

## Related literature

For the crystallographic analysis and functionalization of 6,11-dihydr­oxy-5,12-naphthacenedione, see: Tomura *et al.* (2008 ▶); Ono *et al.* (2009 ▶). For the synthesis of the title compound, see: Ji *et al.* (2006 ▶).
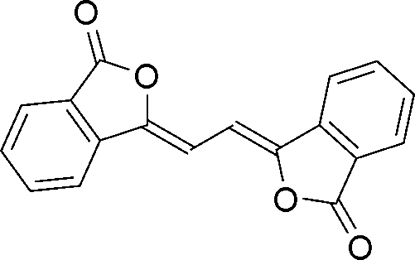


## Experimental

### 

#### Crystal data

C_18_H_10_O_4_
*M* *_r_* = 290.26
Triclinic, 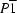

*a* = 6.9030 (13) Å
*b* = 7.0374 (16) Å
*c* = 7.7792 (13) Å
α = 112.190 (5)°
β = 95.696 (2)°
γ = 107.239 (8)°
*V* = 324.53 (11) Å^3^
*Z* = 1
Mo *K*α radiation
μ = 0.11 mm^−1^
*T* = 173 K
1.00 × 0.50 × 0.40 mm

#### Data collection

Rigaku/MSC Mercury CCD diffractometer
Absorption correction: none
2656 measured reflections
1661 independent reflections
1323 reflections with *I* > 2σ(*I*)
*R*_int_ = 0.044

#### Refinement

*R*[*F* ^2^ > 2σ(*F* ^2^)] = 0.066
*wR*(*F* ^2^) = 0.176
*S* = 0.97
1661 reflections
100 parameters
H-atom parameters constrained
Δρ_max_ = 0.35 e Å^−3^
Δρ_min_ = −0.28 e Å^−3^

### 

Data collection: *CrystalClear* (Rigaku/MSC, 2006 ▶); cell refinement: *CrystalClear*; data reduction: *CrystalClear*; program(s) used to solve structure: *SIR2004* (Burla *et al.*, 2005 ▶); program(s) used to refine structure: *SHELXL97* (Sheldrick, 2008 ▶); molecular graphics: *PLATON* (Spek, 2009 ▶) and *Mercury* (Macrae *et al.*, 2006 ▶); software used to prepare material for publication: *WinGX* (Farrugia, 1999 ▶).

## Supplementary Material

Crystal structure: contains datablocks I, global. DOI: 10.1107/S1600536809030888/bt5025sup1.cif

Structure factors: contains datablocks I. DOI: 10.1107/S1600536809030888/bt5025Isup2.hkl

Additional supplementary materials:  crystallographic information; 3D view; checkCIF report

## Figures and Tables

**Table 1:** Hydrogen-bond geometry (Å, °)

*D*—H⋯*A*	*D*—H	H⋯*A*	*D*⋯*A*	*D*—H⋯*A*
C3—H3⋯O2^i^	0.95	2.70	3.459 (2)	137
C5—H5⋯O1^ii^	0.95	2.66	3.406 (2)	136
C6—H6⋯O2^iii^	0.95	2.71	3.4700 (19)	138
C9—H9⋯O2^iii^	0.95	2.55	3.3209 (18)	139

Symmetry codes: (i) 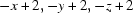; (ii) 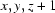; (iii) 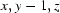.

**Fig. 1.**
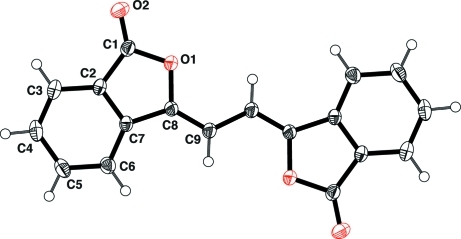
The molecular structure of (I), with atom labels and 50% probability displacement ellipsoids for non-H atoms and H atoms are shown as small spheres of arbitrary radii.

**Fig. 2.**
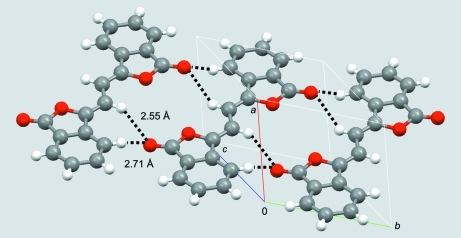
Aggregation along the b axis with C—H···O contacts.

**Fig. 3.**
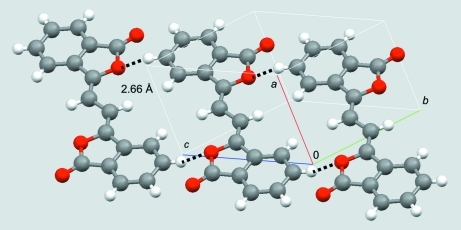
Aggregation along the c axis with C—H···O contacts.

**Fig. 4.**
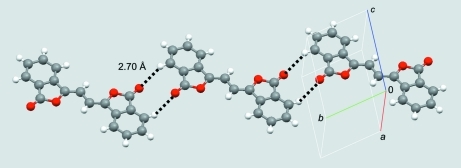
Aggregation along the (021) direction with C—H···O contacts.

**Fig. 5.**
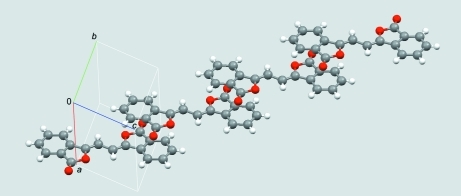
π-Stacking structure with an interplanar distance of 3.43 Å.

## supplementary crystallographic information

### Comment

Through the study of the crystallographic analysis and functionalities of 6,11-dihydroxy-5,12-naphthacenedione (Tomura *et al.*, 2008; Ono *et al.*, 2009), we found the existence of a yellow fluorescent compound as a by-product in the commercially available chemical reagent. After the separation of the by-product, the structure was determined to be the title compound, (I). Although the synthesis of (I) was already reported (Ji *et al.*, 2006), the characterization has not been performed. In this paper, we report the separation, spectral data, and molecular and crystal structures of (I).

The molecular structure of (I) is shown in Fig. 1. The molecule is centrosymmetric and planar with an r.m.s. deviation of 0.031 (1) Å. The distances of the C9—C9 (-*x* + 1, -*y*, -*z* + 1) and C8—C9 bonds are 1.433 (3) and 1.345 (2) Å, respectively, indicating a butadiene skeleton. The bond length of C1—O2 [1.201 (2) Å] is reasonable for carbonyl group. Further, the O1—C1 and O1—C8 bonds result in O—C single bonds with a bond distance of 1.394 (2) Å. These bond distances support the molecular structure of (I).

In the crystal structure, the molecules are linked to each other through intermolecular C—H···O interactions (Table 1). The molecular arrangements shown in Figs. 2 and 3 run stepwise along the *b* and *c* axes, respectively. Fig. 4 also exhibits a stepwise arrangement along the (021) direction with C—H···O contacts. Furthermore, the 3-methyleneisobenzofuran-1(3*H*)-one rings overlap each other to form a π-stacking structure with an interplanar distance of 3.43 Å, as shown in Fig. 5.

### Experimental

The commercially available 6,11-dihydroxy-5,12-naphthacenedione (100 mg, Aldrich) was recrystallized from ethyl acetate (280 ml) to afford pure red crystals of 6,11-dihydroxy-5,12-naphthacenedione (70 mg). The filtrate containing the title compound, (I) was concentrated, and the residue (25 mg) was dissolved in toluene (100 ml). The solution was washed with 1 *M* NaOH solutions (100 ml) ten times over until the NaOH solution was colorless. The organic solution was dried over Na_2_SO_4_ and concentrated to provide a yellow solid of (I) (*ca* 9% yield). Yellow prisms of the title compound, suitable for X-ray analysis were grown from a dichloromethane solution.

### Refinement

All H atoms were placed in geometrically calculated positions, with C—H = 0.95 (aromatic) Å and *U*_iso_(H) = 1.2*U*_eq_(C) (aromatic), and refined using a riding model.

### Crystal data

**Table:**

C_18_H_10_O_4_	*Z* = 1
*M**_r_* = 290.26	*F*(000) = 150
Triclinic, *P*1	*D*_x_ = 1.485 Mg m^−^^3^
Hall symbol: -P 1	Mo *K*α radiation, λ = 0.71073 Å
*a* = 6.9030 (13) Å	Cell parameters from 1131 reflections
*b* = 7.0374 (16) Å	θ = 4.8–30.8°
*c* = 7.7792 (13) Å	µ = 0.11 mm^−^^1^
α = 112.190 (5)°	*T* = 173 K
β = 95.696 (2)°	Prism, yellow
γ = 107.239 (8)°	1.00 × 0.50 × 0.40 mm
*V* = 324.53 (11) Å^3^	

### Data collection

**Table:**

Rigaku/MSC Mercury CCD diffractometer	1323 reflections with *I* > 2σ(*I*)
Radiation source: Rotating Anode	*R*_int_ = 0.044
Graphite Monochromator	θ_max_ = 30.8°, θ_min_ = 3.7°
Detector resolution: 14.62 pixels mm^-1^	*h* = −9→9
φ and ω scans	*k* = −9→5
2656 measured reflections	*l* = −7→10
1661 independent reflections	

### Refinement

**Table:**

Refinement on *F*^2^	Primary atom site location: structure-invariant direct methods
Least-squares matrix: full	Secondary atom site location: difference Fourier map
*R*[*F*^2^ > 2σ(*F*^2^)] = 0.066	Hydrogen site location: inferred from neighbouring sites
*wR*(*F*^2^) = 0.176	H-atom parameters constrained
*S* = 0.97	*w* = 1/[σ^2^(*F*_o_^2^) + (0.1211*P*)^2^] where *P* = (*F*_o_^2^ + 2*F*_c_^2^)/3
1661 reflections	(Δ/σ)_max_ < 0.001
100 parameters	Δρ_max_ = 0.35 e Å^−^^3^
0 restraints	Δρ_min_ = −0.28 e Å^−^^3^

### Special details

**Table:**

Experimental. IR (KBr, cm^-1^): 1784, 1645, 1472, 1343, 1281, 1073, 974, 766, 689; ^1^H NMR (CDCl_3_, δ p.p.m.): 6.86 (s, 2H), 7.60 (t, J = 7.6 Hz, 2H), 7.77 (t, J = 7.6 Hz, 2H), 7.81 (d, J = 7.6 Hz, 2H), 7.96 (d, J = 7.6 Hz, 2H); MS (EI): m/*z* 290 (*M*^+^), 104; UV-vis (CH_2_Cl_2_, nm): 406, 383.
Geometry. All e.s.d.'s (except the e.s.d. in the dihedral angle between two l.s. planes) are estimated using the full covariance matrix. The cell e.s.d.'s are taken into account individually in the estimation of e.s.d.'s in distances, angles and torsion angles; correlations between e.s.d.'s in cell parameters are only used when they are defined by crystal symmetry. An approximate (isotropic) treatment of cell e.s.d.'s is used for estimating e.s.d.'s involving l.s. planes.
Refinement. Refinement of *F*^2^ against ALL reflections. The weighted *R*-factor *wR* and goodness of fit *S* are based on *F*^2^, conventional *R*-factors *R* are based on *F*, with *F* set to zero for negative *F*^2^. The threshold expression of *F*^2^ > σ(*F*^2^) is used only for calculating *R*-factors(gt) *etc*. and is not relevant to the choice of reflections for refinement. *R*-factors based on *F*^2^ are statistically about twice as large as those based on *F*, and *R*- factors based on ALL data will be even larger.

### Fractional atomic coordinates and isotropic or equivalent isotropic displacement parameters (Å^2^)

**Table:**

	*x*	*y*	*z*	*U*_iso_*/*U*_eq_	
C1	0.7382 (2)	0.6230 (2)	0.8120 (2)	0.0242 (4)	
C2	0.7695 (2)	0.5873 (2)	0.9849 (2)	0.0216 (4)	
C3	0.8463 (2)	0.7402 (2)	1.1749 (2)	0.0285 (4)	
H3	0.8898	0.8939	1.2100	0.034*	
C4	0.8568 (2)	0.6597 (3)	1.3105 (2)	0.0320 (4)	
H4	0.9049	0.7593	1.4421	0.038*	
C5	0.7974 (2)	0.4324 (3)	1.2568 (2)	0.0310 (4)	
H5	0.8079	0.3814	1.3531	0.037*	
C6	0.7236 (2)	0.2804 (2)	1.0665 (2)	0.0267 (4)	
H6	0.6859	0.1271	1.0308	0.032*	
C7	0.7070 (2)	0.3616 (2)	0.9299 (2)	0.0204 (3)	
C8	0.6284 (2)	0.2534 (2)	0.7233 (2)	0.0207 (3)	
C9	0.5404 (2)	0.0392 (2)	0.6005 (2)	0.0223 (4)	
H9	0.5313	−0.0668	0.6501	0.027*	
O1	0.65059 (16)	0.41594 (15)	0.65779 (15)	0.0248 (3)	
O2	0.77382 (19)	0.78743 (17)	0.78771 (18)	0.0364 (4)	

### Atomic displacement parameters (Å^2^)

**Table:**

	*U*^11^	*U*^22^	*U*^33^	*U*^12^	*U*^13^	*U*^23^
C1	0.0231 (7)	0.0198 (7)	0.0242 (8)	0.0061 (6)	0.0010 (6)	0.0061 (6)
C2	0.0187 (6)	0.0193 (7)	0.0207 (7)	0.0044 (5)	0.0025 (5)	0.0046 (5)
C3	0.0263 (8)	0.0231 (7)	0.0245 (8)	0.0056 (6)	0.0027 (6)	0.0016 (6)
C4	0.0296 (8)	0.0343 (9)	0.0180 (8)	0.0048 (7)	0.0024 (6)	0.0027 (6)
C5	0.0287 (8)	0.0389 (9)	0.0197 (8)	0.0053 (7)	0.0045 (6)	0.0125 (7)
C6	0.0252 (7)	0.0270 (7)	0.0234 (8)	0.0050 (6)	0.0038 (6)	0.0101 (6)
C7	0.0176 (7)	0.0207 (7)	0.0188 (7)	0.0050 (5)	0.0031 (5)	0.0059 (5)
C8	0.0209 (7)	0.0205 (7)	0.0185 (7)	0.0070 (5)	0.0017 (5)	0.0072 (5)
C9	0.0221 (7)	0.0197 (7)	0.0208 (8)	0.0058 (5)	0.0009 (5)	0.0066 (6)
O1	0.0294 (6)	0.0188 (5)	0.0205 (6)	0.0055 (4)	−0.0005 (4)	0.0063 (4)
O2	0.0436 (7)	0.0221 (6)	0.0382 (7)	0.0073 (5)	−0.0006 (5)	0.0136 (5)

### Geometric parameters (Å, °)

**Table:**

C1—O2	1.2007 (18)	C5—C6	1.388 (2)
C1—O1	1.3935 (17)	C5—H5	0.9500
C1—C2	1.465 (2)	C6—C7	1.393 (2)
C2—C3	1.389 (2)	C6—H6	0.9500
C2—C7	1.3914 (18)	C7—C8	1.4543 (19)
C3—C4	1.378 (2)	C8—C9	1.3447 (19)
C3—H3	0.9500	C8—O1	1.3944 (17)
C4—C5	1.405 (2)	C9—C9^i^	1.433 (3)
C4—H4	0.9500	C9—H9	0.9500
			
O2—C1—O1	120.77 (15)	C4—C5—H5	119.2
O2—C1—C2	132.22 (15)	C5—C6—C7	117.42 (14)
O1—C1—C2	107.01 (12)	C5—C6—H6	121.3
C3—C2—C7	122.43 (14)	C7—C6—H6	121.3
C3—C2—C1	129.48 (14)	C2—C7—C6	120.35 (14)
C7—C2—C1	108.08 (13)	C2—C7—C8	107.24 (13)
C4—C3—C2	117.22 (14)	C6—C7—C8	132.40 (13)
C4—C3—H3	121.4	C9—C8—O1	120.45 (13)
C2—C3—H3	121.4	C9—C8—C7	131.48 (13)
C3—C4—C5	120.95 (14)	O1—C8—C7	108.04 (11)
C3—C4—H4	119.5	C8—C9—C9^i^	124.02 (16)
C5—C4—H4	119.5	C8—C9—H9	118.0
C6—C5—C4	121.59 (15)	C9^i^—C9—H9	118.0
C6—C5—H5	119.2	C1—O1—C8	109.58 (12)
			
O2—C1—C2—C3	2.1 (3)	C5—C6—C7—C2	2.1 (2)
O1—C1—C2—C3	−177.95 (14)	C5—C6—C7—C8	−176.59 (13)
O2—C1—C2—C7	−177.93 (16)	C2—C7—C8—C9	−176.24 (14)
O1—C1—C2—C7	2.00 (15)	C6—C7—C8—C9	2.6 (3)
C7—C2—C3—C4	−0.7 (2)	C2—C7—C8—O1	1.63 (15)
C1—C2—C3—C4	179.22 (13)	C6—C7—C8—O1	−179.57 (13)
C2—C3—C4—C5	1.7 (2)	O1—C8—C9—C9^i^	−1.6 (3)
C3—C4—C5—C6	−0.8 (2)	C7—C8—C9—C9^i^	176.07 (16)
C4—C5—C6—C7	−1.1 (2)	O2—C1—O1—C8	178.96 (12)
C3—C2—C7—C6	−1.2 (2)	C2—C1—O1—C8	−0.98 (16)
C1—C2—C7—C6	178.83 (12)	C9—C8—O1—C1	177.79 (11)
C3—C2—C7—C8	177.76 (13)	C7—C8—O1—C1	−0.36 (16)
C1—C2—C7—C8	−2.20 (15)		

Symmetry codes: (i) −*x*+1, −*y*, −*z*+1.

### Hydrogen-bond geometry (Å, °)

**Table:**

*D*—H···*A*	*D*—H	H···*A*	*D*···*A*	*D*—H···*A*
C3—H3···O2^ii^	0.95	2.70	3.459 (2)	137
C5—H5···O1^iii^	0.95	2.66	3.406 (2)	136
C6—H6···O2^iv^	0.95	2.71	3.4700 (19)	138
C9—H9···O2^iv^	0.95	2.55	3.3209 (18)	139

Symmetry codes: (ii) −*x*+2, −*y*+2, −*z*+2; (iii) *x*, *y*, *z*+1; (iv) *x*, *y*−1, *z*.
